# Post-Transplant Extracorporeal Membrane Oxygenation for Severe Primary Graft Dysfunction to Support the Use of Marginal Donor Hearts

**DOI:** 10.3389/ti.2022.10176

**Published:** 2022-03-10

**Authors:** Yasuhiro Shudo, Aiman Alassar, Hanjay Wang, Bharathi Lingala, Hao He, Yuanjia Zhu, William Hiesinger, John W. MacArthur, Jack H. Boyd, Anson M. Lee, Maria Currie, Y. Joseph Woo

**Affiliations:** Department of Cardiothoracic Surgery, Stanford University School of Medicine, Stanford, CA, United States

**Keywords:** outcomes, heart transplantation, ECMO, primary graft dysfunction, marginal donor heart

## Abstract

Severe primary graft dysfunction (PGD) is the leading cause of early postoperative mortality following orthotopic heart transplantation (OHT). Veno-arterial extracorporeal membrane oxygenation (VA-ECMO) has been used as salvage therapy. This study aimed to evaluate the outcomes in adult OHT recipients who underwent VA-ECMO for severe PGD. We retrospectively reviewed 899 adult (≥18 years) patients who underwent primary OHT at our institution between 1997 and 2017. Recipients treated with VA-ECMO (19, 2.1%) exhibited a higher incidence of previous cardiac surgery (*p* = .0220), chronic obstructive pulmonary disease (*p* = .0352), and treatment with a calcium channel blocker (*p* = .0018) and amiodarone (*p* = .0148). Cardiopulmonary bypass (*p* = .0410) and aortic cross-clamp times (*p* = .0477) were longer in the VA-ECMO cohort and they were more likely to have received postoperative transfusion (*p* = .0013); intra-aortic balloon pump (IABP, *p* < .0001), and reoperation for bleeding or tamponade (*p* < .0001). The 30-day, 1-year, and overall survival after transplantation of non-ECMO patients were 95.9, 88.8, and 67.4%, respectively, compared to 73.7, 57.9, and 47.4%, respectively in the ECMO cohort. Fourteen (73.7%) of the ECMO patients were weaned after a median of 7 days following OHT (range: 1–12 days). Following OHT, VA-ECMO may be a useful salvage therapy for severe PGD and can potentially support the usage of marginal donor hearts.

## Graphical Abstract

Post-transplant extracorporeal membrane oxygenation for severe primary graft dysfunction to support the use of marginal donor hearts.
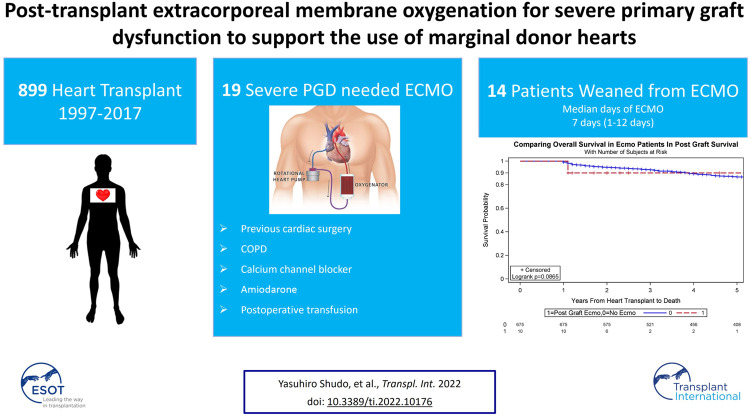

## Introduction

Heart disease is the leading cause of death in the United States, and medically refractory heart failure represents end-stage heart disease (1). We are currently faced with a plethora of patients suffering from heart failure. Many treatments have been developed for patients with end-stage heart failure, among which orthotopic heart transplantation (OHT) remains the gold standard (2). However, primary graft dysfunction (PGD) is a devastating complication, and the associated 30-day mortality rate is as high as 30% (3–5). PGD is diagnosed within 24 h after OHT and is distinct from secondary graft dysfunction where there is a discernible cause such as hyperacute rejection, pulmonary hypertension, or known surgical complications (6). There are several possible treatment options for managing PGD, such as inotropes, intra-aortic balloon pump (IABP), and mechanical circulatory assist, among others. According to the International Society for Heart and Lung Transplantation (ISHLT) Registry consensus statement (6), the most severe form of PGD was defined as the requirement of mechanical circulatory assistance for treatment.

Although over 20,000 patients may benefit from OHT per year, only 3,000 will receive a new heart, with a waitlist mortality of 10.7 deaths per 100,000 waitlist-years (7). Due to the persistent and worsening shortage of available donor hearts, we have previously proposed alternative approaches to maximize organ allocation, including repairing the donor’s valvular heart disease (8), harvesting donor hearts from more distant locations and accepting longer cold ischemic time (9), as well as utilizing hearts from obese donors (10). Despite growing evidence supporting the safety of using these marginal organs, there are concerns regarding PGD following OHT with marginal hearts.

Veno-arterial extracorporeal membrane oxygenation (VA-ECMO) is a versatile mechanical circulatory support technique that may be used as salvage therapy for patients with low-output post-cardiotomy syndrome. In the context of OHT, VA-ECMO represents an increasingly common therapeutic option for post-transplant recipients with severely depressed postoperative cardiac output and dysfunction (3–5). Therefore, this study aimed to review the outcomes of adult heart transplant recipients who underwent VA-ECMO for severe PGD.

## Methods

For confidentiality reasons, the data and study materials will not be made available to other researchers for purposes of reproducing the results.

### Patient Selection

We retrospectively reviewed all patients who underwent OHT at Stanford University Hospital between January 1997 and December 2017 (*n* = 1,181).

**FIGURE 1:**
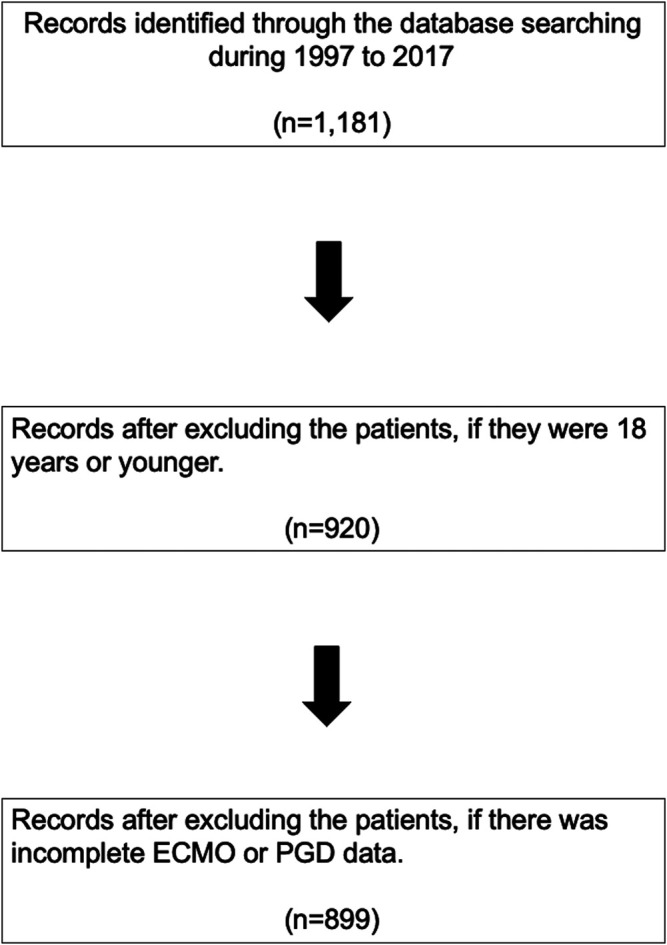
PRISMA flow diagram. ECMO, extracorporeal membrane oxygenation; PGD, primary graft dysfunction.

The exposure of interest was postoperative VA-ECMO usage within 30 days of OHT due to severe PGD. Patients were excluded if they were below 18 years old (*n* = 261), or if there was incomplete post-OHT ECMO data (*n* = 21, Figure 1). The patients were assigned to two groups based on the requirement of VA-ECMO to manage severe PGD following OHT.

Information obtained from our institutional database included donor characteristics (age, sex, height, body weight, body mass index), past medical history (diabetes, hypertension, tobacco use, hepatitis C), donor’s left ventricular ejection fraction, recipient baseline characteristics (age, sex, height, body weight, and body mass index), past medical history (diabetes, hypertension, hyperlipidemia, hemodialysis, chronic obstructive pulmonary disease, history of cerebrovascular accident), etiology of heart failure, total waiting time, and preoperative life support (hospitalization, inotropic support, ventilator support, IABP, ECMO, durable ventricular assist device [VAD]), preoperative medication, and allograft ischemic time.

The primary outcomes were 30-day, 1-year, and overall mortality, which were defined as patient death post transplantation. Studies involving this dataset have been exempted from review by the Institutional Review Board of Stanford University School of Medicine.

### Statistical Analysis

In the descriptive analyses of the study, continuous variables were presented as means ± standard deviation and compared to the mean differences between groups by analysis of variance (ANOVA). The *χ* ^2^ test was used to evaluate the association between the categorical variables. Survival curves were constructed using the Kaplan-Meier method, stratified over post-transplant ECMO usage, and were tested using the log-rank test. Exact matching with risk adjustment for confounders was performed to identify patients who did not undergo ECMO after transplantation but who had similar essential characteristics as those who received post-transplant VA-ECMO support (4). The matching criteria for this study were: transplant year ±5 years, recipient age ±4 years, recipient gender, recipient history of prior cardiac surgery, and recipient preoperative life support (inotropic support). Matching criteria were applied sequentially to produce two matched cohorts containing all the possible pairings. The endpoints were then compared between the two matched cohorts. For all analyses, *p*-values <.05, were considered statistically significant. All analyses were performed using SAS version 9.4 (SAS Institute Inc. NC, United States).

## Results

A total of 899 adult (≥18 years) primary OHT patients who fulfilled the study entry criteria were identified. The cohorts differed in demographic and preoperative clinical characteristics, depending on the requirement for post-transplant VA-ECMO. Nineteen patients (2.1%) received VA-ECMO support in the very early post-transplant period due to severe PGD, and 880 patients (97.9%) did not receive VA-ECMO.

### Recipient Characteristics

Recipient characteristics stratified by recipient post-transplant ECMO use are shown in Table 1.

**TABLE 1:** Recipient characteristics stratified by recipient post-transplant ECMO usage.

	*N* ^a^	Without ECMO	*N* ^a^	With ECMO	*N* ^a^	Total	*p* value
Age (y)	880	51.55 ± 12.92 [55 (45, 61)]	19	48.26 ± 12.45 [49 (40, 58)]	899	51.48 ± 12.92 [54 (45, 61)]	.1058
Gender, male, *n* (%)	879	649 (73.83%)	19	15 (78.95%)	898	664 (73.94%)	.7938
Height (cm)	846	166.14 ± 35.19 [172.7 (165.1, 180.3)]	19	139.96 ± 74 [172.7 (160, 182.9)]	865	165.56 ± 36.6 [172.7 (165.1, 180.3)]	.9809
Body weight (kg)	846	78.69 ± 17.04 [78 (66.4, 89.8)]	19	81.49 ± 30.91 [82.5 (62.59, 102.5)]	865	78.75 ± 17.43 [78 (66.4, 89.8)]	.325
Body mass index (kg/m^2^)	835	26.19 ± 4.91 [25.6 (22.8, 29)]	18	27.89 ± 6.94 [26 (24.7, 34.3)]	853	26.22 ± 4.96 [25.6 (22.8, 29)]	.6293
Past medical history							
Diabetes mellitus, *n* (%)	880	278 (31.59%)	19	7 (36.84%)	899	285 (31.7%)	.624
Hypertension, *n* (%)	880	388 (44.09%)	19	11 (57.89%)	899	399 (44.38%)	.2508
Hyperlipidemia, *n* (%)	880	346 (39.32%)	19	7 (36.84%)	899	353 (39.27%)	1
On hemodialysis, *n* (%)	880	35 (3.98%)	19	2 (10.53%)	899	37 (4.12%)	.182
COPD, *n* (%)	880	86 (9.77%)	19	5 (26.32%)	899	91 (10.12%)	.0352
History of CVA, *n* (%)	880	36 (4.09%)	19	2 (10.53%)	899	38 (4.23%)	.1898
Tobacco usage, *n* (%)	880	209 (23.75%)	19	8 (42.11%)	899	217 (24.14%)	.0983
Etiology of heart failure							
Non-ischemic cardiomyopathy, *n* (%)	879	290 (32.99%)	19	2 (10.53%)	898	292 (32.52%)	.1872
Ischemic cardiomyopathy, *n* (%)	879	234 (26.62%)	19	6 (31.58%)	898	240 (26.73%)	
Congenital heart disease, *n* (%)	879	71 (8.08%)	19	4 (21.05%)	898	75 (8.35%)	
Restrictive heart disease, *n* (%)	879	62 (7.05%)	19	1 (5.26%)	898	63 (7.02%)	
Hypertrophic cardiomyopathy, *n* (%)	879	57 (6.48%)	19	1 (5.26%)	898	58 (6.46%)	
Valvular heart disease, *n* (%)	879	19 (2.16%)	19	0 (0%)	898	19 (2.12%)	
Familial cardiomyopathy, *n* (%)	879	40 (4.55%)	19	1 (5.26%)	898	41 (4.57%)	
Repeat heart transplantation, *n* (%)	879	39 (4.44%)	19	1 (5.26%)	898	40 (4.45%)	
Total waitlist time (years)	841	131.33 ± 250.52 [47 (16, 134)]	17	215.29 ± 211.36 [138 (43, 314)]	858	132.99 ± 249.97 [47 (16, 138)]	.086
Previous cardiac surgery, *n* (%)	880	274 (31.14%)	19	11 (57.89%)	899	285 (31.7%)	.022
Pre-operative life support, *n* (%)							
Hospitalization, *n* (%)	866	311 (35.91%)	18	6 (33.33%)	884	317 (35.86%)	1
Inotropic support, *n* (%)	842	368 (43.71%)	19	13 (68.42%)	861	381 (44.25%)	.0367
Ventilator support, *n* (%)	833	92 (11.04%)	17	3 (17.65%)	850	95 (11.18%)	.4243
IABP, *n* (%)	833	10 (1.2%)	17	1 (5.88%)	850	11 (1.29%)	.2003
ECMO, *n* (%)	833	1 (0.12%)	17	0 (0%)	850	1 (0.12%)	1
Durable VAD, *n* (%)	854	172 (20.14%)	18	5 (27.78%)	872	177 (20.3%)	.3855
Pre-operative medication, *n* (%)							
Beta blocker, *n* (%)	756	214 (28.31%)	16	7 (43.75%)	772	221 (28.63%)	.1746
Calcium channel blocker, *n* (%)	740	41 (5.54%)	16	5 (31.25%)	756	46 (6.08%)	.0018
Angiotensin receptor blocker, *n* (%)	763	181 (23.72%)	16	3 (18.75%)	779	184 (23.62%)	.7743
Angiotensin converting enzyme-inhibitor, *n* (%)	752	113 (15.03%)	16	2 (12.5%)	768	115 (14.97%)	1
Aspirin, *n* (%)	765	268 (35.03%)	16	6 (37.5%)	781	274 (35.08%)	.7979
Plavix, *n* (%)	495	35 (7.07%)	16	1 (6.25%)	511	36 (7.05%)	1
Anticoagulation (Warfarin, heparin), *n* (%)	767	387 (50.46%)	16	6 (37.5%)	783	393 (50.19%)	.3257
Lasix, *n* (%)	544	204 (37.5%)	16	5 (31.25%)	560	209 (37.32%)	.7945
Spironolactone, *n* (%)	750	234 (31.2%)	16	9 (56.25%)	766	243 (31.72%)	.0531
Amiodarone, *n* (%)	734	283 (38.56%)	12	9 (75%)	746	292 (39.14%)	.0148
Digoxin, *n* (%)	756	175 (23.15%)	16	4 (25%)	772	179 (23.19%)	.7721
Pre-operative data							
White blood cell count (×1,000/ml)	761	7.85 ± 2.88 [7.3 (5.9, 9.1)]	14	8.93 ± 3.28 [9.05 (6.4, 11.4)]	775	7.87 ± 2.89 [7.3 (5.9, 9.1)]	.2777
Hemoglobin (g/dl)	737	11.57 ± 2.11 [11.5 (10.1, 13)]	14	12.14 ± 2.62 [12.85 (10, 13.7)]	751	11.58 ± 2.12 [11.5 (10.1, 13)]	.2786
Platelet (×1,000/ml)	763	223.42 ± 89.1 [207 (165, 261)]	14	190.14 ± 73.76 [181.5 (144, 216)]	777	222.82 ± 88.92 [207 (165, 260)]	.1058
Sodium (mmol/L)	585	134.1 ± 4.92 [135 (131, 137)]	11	136.45 ± 5.11 [135 (134, 137)]	596	134.15 ± 4.93 [135 (131, 137)]	.6776
Blood urea nitrogen (mg/dl)	772	29.76 ± 18.41 [24.5 (18, 35)]	15	26.6 ± 12.77 [25 (14, 38)]	787	29.7 ± 18.32 [25 (18, 35)]	.804
Creatinine (mg/dl)	772	1.52 ± 0.96 [1.3 (1, 1.7)]	15	1.56 ± 0.66 [1.39 (1, 2.1)]	787	1.52 ± 0.96 [1.3 (1, 1.7)]	.7849
Total bilirubin (mg/dl)	539	1.26 ± 1.32 [1 (0.6, 1.5)]	10	1.1 ± 1.55 [0.6 (0.4, 1)]	549	1.26 ± 1.32 [1 (0.6, 1.5)]	.1949
Aspartate transaminase (U/L)	550	46.01 ± 67.33 [30 (23, 43)]	10	47.3 ± 47.32 [31.5 (21, 46)]	560	46.03 ± 67 [30 (23, 43)]	1
Alanine transaminase (U/L)	540	57.83 ± 148.8 [34 (24, 48)]	9	49.33 ± 35.48 [38 (31, 48)]	549	57.69 ± 147.64 [34 (24, 48)]	.7308
Albumin (g/dl)	548	3.42 ± 0.63 [3.4 (3, 3.9)]	10	3.52 ± 0.52 [3.6 (3, 3.8)]	558	3.42 ± 0.63 [3.45 (3, 3.9)]	.5237
INR	544	1.89 ± 0.89 [1.6 (1.2, 2.4)]	9	1.68 ± 0.64 [1.4 (1.1, 2.3)]	553	1.89 ± 0.89 [1.6 (1.2, 2.4)]	.7375

ECMO, extra corporealmembrane oxygenation. COPD, chronic obstructive pulmonary disease. CVA, cerebrovascular accident. IABP, Intra-aortic baloon pump. VAD, ventricular assist device. INR, international normalized ratio.

a *N*, available number of patients.

The mean age of all recipients was 51.5 ± 12.9 years. A total of 73.9% of recipients were male, and the mean body mass index was 26.2 ± 5.0 kg/m^2^. Overall, 31.7% of recipients were diabetic, 44.4% were hypertensive, 39.3% had a history of hyperlipidemia, 24.1% had a history of cigarette use, 10.1% had a history of COPD, and 4.1% were on hemodialysis.

The prevalence of COPD in recipients undergoing ECMO after OHT (26.3%) was significantly higher than that in recipients who did not undergo ECMO after OHT (9.8%), *p* = .0352. In addition, the prevalence of previous cardiac surgery was significantly greater among recipients in the post-transplant ECMO group (57.9%) than among recipients without post-transplant ECMO (31.1%), *p* = .022. The percentages of patients receiving a calcium channel blocker (31.3% vs. 5.5%, *p* = .0018) and amiodarone (75.0% vs. 38.6%, *p* = .0148) were also significantly higher in the ECMO cohort compared to the non-ECMO cohort.

Mechanical circulatory support usage before OHT was not significantly different between the two groups (IABP, ECMO, and durable VAD; *p* = .2003, 1, and .3855, respectively). Similarly, the proportion of patients admitted to the intensive care unit (ICU) prior to OHT was not significantly different between the two groups. These results suggest that post-transplant ECMO was utilized independently and was not associated with the recipient’s preoperative clinical status.

### Donor Characteristics

Donor characteristics stratified by post-transplant VA-ECMO use are shown in Table 2. The mean age of all donors was 33.0 ± 12.4 years. A total of 73.5% of donors were male, and the mean body mass index was 26.7 ± 5.5 kg/m^2^. Overall, 2.4% of donors were diabetic, 13.0% were hypertensive, and 21.9% had a history of cigarette use. The incidence of hepatitis C positive donors was extremely low (1.0%). The left ventricular ejection fraction was excellent in both groups. There were no significant differences in the donor baseline characteristics between the two groups.

**TABLE 2:** Donor characteristics stratified by recipient post-transplant ECMO usage.

Donors’ characteristics	*N* ^a^	Without ECMO	*N* ^a^	With ECMO	*N* ^a^	Total	*p* value
Age (y)	880	32.98 ± 12.4 [31 (22, 43)]	19	35.84 ± 12.73 [40 (22, 45)]	899	33.04 ± 12.4 [32 (22, 43)]	.4829
Gender, male, *n* (%)	856	628 (73.36%)	18	14 (77.78%)	874	642 (73.46%)	.7931
Height (cm)	856	174.32 ± 9.7 [175 (168, 181)]	18	176.96 ± 8.78 [177 (171, 183)]	874	174.38 ± 9.69 [175 (168, 181)]	.244
Body weight (kg)	856	81.3 ± 18.47 [79 (69, 90.7)]	18	77.34 ± 22.62 [77.5 (61, 81.5)]	874	81.22 ± 18.56 [79 (69, 90.2)]	.5091
Body mass index (kg/m^2^)	856	26.72 ± 5.48 [25.9 (22.7, 29.4)]	18	24.66 ± 7.14 [23.25 (19.9, 26.6)]	874	26.68 ± 5.52 [25.9 (22.7, 29.3)]	.1524
Donor’s ejection fraction (%)	600	64.85 ± 11.08 [64.73 (60, 71.76)]	14	64.62 ± 11.47 [64.97 (59, 72.96)]	614	64.85 ± 11.08 [64.73 (60, 71.83)]	1
Past medical history							
Diabetes mellitus, *n* (%)	849	21 (2.47%)	18	0 (0%)	867	21 (2.42%)	1
Hypertension, *n* (%)	845	109 (12.9%)	17	3 (17.65%)	862	112 (12.99%)	.4751
Tobacco usage, *n* (%)	834	184 (22.06%)	17	2 (11.76%)	851	186 (21.86%)	.3902
Hepatitis C positive, n (%)	823	8 (0.97%)	19	0 (0%)	842	8 (0.95%)	1

ECMO, extracorporeal membrane oxygenation.

a *N*, available number of patients.

### Operative Variables

Operative variables stratified by post-transplant VA ECMO use are shown in Table 3. Cardiopulmonary bypass (209.7 ± 59.1 vs. 167.2 ± 52.8 min, *p* = .041) and aortic cross clamp times (125.4 ± 44.9 vs. 102.2 ± 44.5 min, *p* = .0477) were longer in the post-transplant ECMO cohort. There were no significant differences between recipients with ECMO (232.1 ± 69.0 min) and those without ECMO (219.9 ± 56.6 min, *p* = .2444) regarding the allograft ischemic time.

**TABLE 3:** Operative measures stratified by recipient post-transplant ECMO usage, before and after exact matching.

Operative Measure	Before matching					After matching^a^				
	Without ECMO		With ECMO		*p*-value	Without ECMO		With ECMO		*p*-value
	*N* ^b^	Estimate	*N* ^b^	Estimate		*N* ^b^	Estimate	*N* ^b^	Estimate	
Cardiopulmonary bypass time (minutes)										
Mean ± SD	768	167.15 ± 52.78	15	209.73 ± 59.14	.0041	57	179.58 ± 48.48	15	209.73 ± 59.14	.3873
Median (IQR)		157 (133, 189)		193 (173, 286)			173 (143, 215)		193 (173, 286)	
Aortic cross clamp time (minutes)										
Mean ± SD	599	102.21 ± 44.54	13	125.38 ± 44.92	.0477	43	112.02 ± 28.49	13	125.38 ± 44.92	.1168
Median (IQR)		95 (80, 115)		122 (107, 136)			103 (92, 138)		122 (107, 136)	
Allograft ischemic time (minutes)										
Mean ± SD	862	219.93 ± 56.61	19	232.1 ± 69	.2444	63	222.2 ± 52.67	19	232.1 ± 69	.9273
Median (IQR)		216 (186, 252)		228 (204, 282)			228 (198, 246)		228 (204, 282)	
Transfusion										
Intraoperative, *n* (%)	553	286 (51.72 %)	16	9 (56.25 %)	.8030	52	39 (75 %)	16	9 (56.25 %)	.2098
Postoperative, *n* (%)	413	222 (53.75 %)	16	15 (93.75 %)	.0013	52	30 (57.69 %)	16	15 (93.75 %)	.0071
Distance organ travelled (miles)										
Mean ± SD	769	140.87 ± 160.06	17	157.24 ± 203.91	.8062	58	120.41 ± 130.65	17	157.24 ± 203.91	.4474
Median (IQR)		81 (25, 168)		51 (31, 254)			119 (23, 147)		51 (31, 254)	
Transplant year										
Median (IQR)	880	2,008 (2,003, 2,014)	19	2,015 (2,012, 2,016)	.0020	63	2,014 (2,010, 2,016)	19	2,015 (2,012, 2,016)	.2365
Postoperative IABP										
*n* (%)	805	33 (4.1%)	16	9 (56.25%)	<.0001	57	4 (7.02%)	16	9 (56.25%)	<.0001
Postoperative VA ECMO										
*n* (%)	876	0 (0%)	19	19 (100%)	N/A^a^	63	0 (0%)	19	19 (100%)	N/A^a^
Postoperative VV ECMO										
*n* (%)	876	0 (0%)	19	4 (21.05%)	N/A^a^	63	0 (0%)	19	4 (21.05%)	N/A^a^
Reoperation for bleeding or tamponade										
*n* (%)	826	61 (7.38%)	16	13 (81.25%)	<.0001	59	8 (13.56%)	16	13 (81.25%)	.0022
Multiorgan transplant										
*n* (%)	813	53 (6.52%)	16	0 (0%)	.6167	0	0 (0%)	16	0 (0%)	N/A^a^

a Patients were matched on Transplant Year (±5 years), Recipient’s Age (±4 years old), Recipient’s Gender, Recipient’s History of Prior Cardiac Surgery, and Recipient’s Preoperative Life Support (inotropic support) with those with ECMO.

b Available number of patients.

c Statistic is not applicable. ECMO, extracorporeal membrane oxygenation. IABP, intra-aortic baloon pump.

The percentage of postoperative transfusion was greater in the post-transplant ECMO group (93.8% vs. 53.8%, *p* = .0013). Similarly, the incidence of reoperation for bleeding or tamponade was greater in the post-transplant ECMO cohort (81.3% vs. 7.4%, *p* < .0001). These results suggest that significant postoperative transfusion and bleeding may cause hemodynamic instability, leading to the requirement for ECMO.

Interestingly, the distance of donor organ travel was similar between the groups (157.2 ± 203.9 miles for recipients with ECMO, compared to 140.9 ± 160.1 miles for those without ECMO, *p* = .8062). There were no multiorgan transplant recipients in the post-transplant ECMO cohort, whereas 6.5% of recipients in the non-ECMO cohort received multiorgan transplants.

### Outcomes

The frequency of postoperative pneumonia (31.6% vs. 7.4%, *p* = .0023) and renal failure requiring dialysis (68.4% vs. 14.2%, *p* < .0001) were significantly higher in the ECMO cohort. Length of hospital stay (49.5 ± 57.8 vs. 20.8 ± 24.4 days, *p* = .0002) and ICU stay (37.1 ± 45.6 vs. 8.8 ± 12.7 days, *p* = .0001) were significantly longer in the post-transplant ECMO cohort.

**FIGURE 2:**
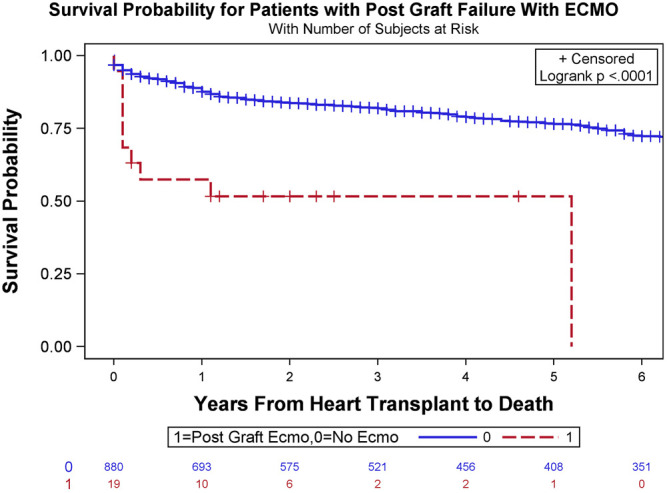
Overall survival Kaplan-Meier estimates stratified based on the requirement of veno-arterial extracorporeal membrane oxygenation (VA-ECMO) to manage severe primary graft dysfunction (PGD) following orthotopic heart transplant (OHT) (log-rank test, *p* < .0001).

In the entire cohort, the 30-day, 1-year, and overall survival rates after transplantation were 95.9, 88.8, and 67.4%, respectively. In the ECMO cohort, the 30-day, 1-year, and overall survival rates after transplantation were 73.7, 57.9, and 47.4%, respectively. To assess the effect of post-transplant ECMO usage on survival, time-to-event survival analyses were conducted. The *p*-value of the log-rank tests on the Kaplan-Meier survival estimations of the two groups was <.0001 for overall survival (Figure 2). The odds ratios of 1-year mortality were 5.737 for the unadjusted analysis and 5.544 for the adjusted analysis (*p* = .0002 and .0004, respectively). Unadjusted and adjusted odds ratios for overall survival were 2.295 and 2.269, respectively, although these differences did not reach statistical significance (*p* = .074 and .0784, respectively).

**FIGURE 3:**
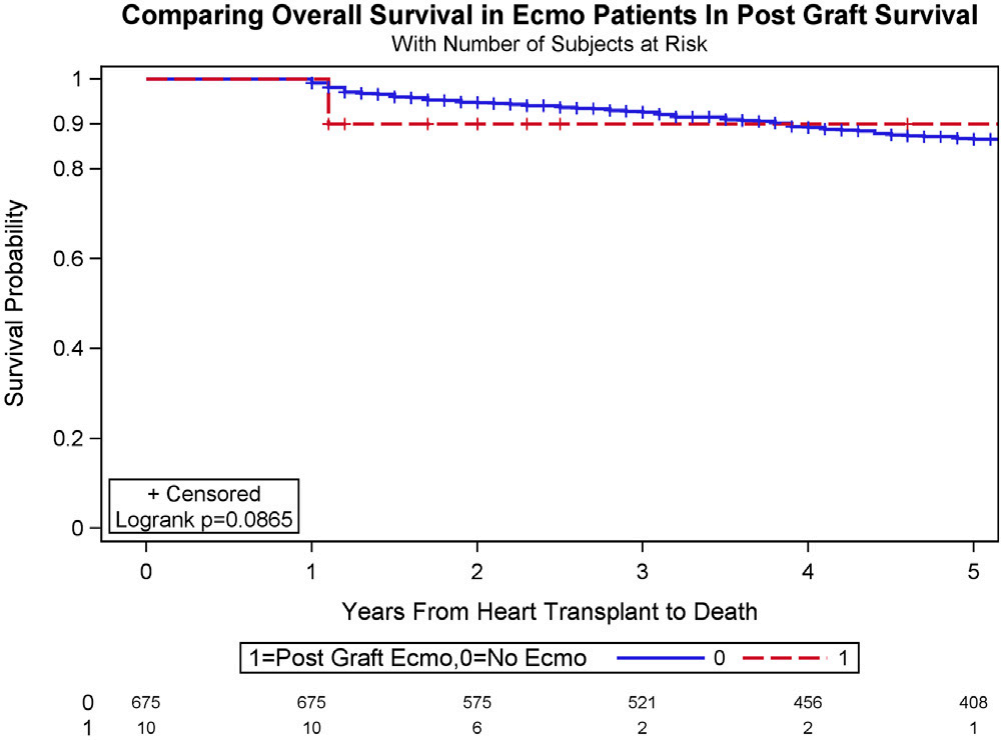
Conditional survival, defined as survival for recipients who survive for at least 1 year after surgery, was 90.0% and 90.0% at 3 years and 5 years in the recipients who underwent veno-arterial extracorporeal membrane oxygenation (VA-ECMO), and 92.6% and 86.5% at 3 years and 5 years in the recipients who did not undergo VA-ECMO (log-rank test, *p* = .0865).

Interestingly, conditional survival, defined as survival for recipients who survived for at least 1 year after surgery, was 92.6% and 86.5% at 3 years and 5 years in the cohort with ECMO, and 90.0% and 90.0% at 3 years and 5 years in the cohort without ECMO (log-rank test, *p* = .0865; Figure 3).

Among the 19 patients with post-transplant ECMO, 14 (73.7%) were weaned from ECMO at a median duration of 7 days following OHT (range: 1–2 days).

### Outcomes After Exact Matching Analysis

Of the 899 recipients in this study, 82 were successfully matched based on several important factors, using the exact matching algorithm previously described (without ECMO, *n* = 63; with ECMO, *n* = 19). In the matched cohort, the mean age for adult primary OHT was 49.1 years old. In total, 68 recipients (82.9%) were men. There were no significant differences in the recipient or donor baseline characteristics between the two matched cohorts.

**FIGURE 4:**
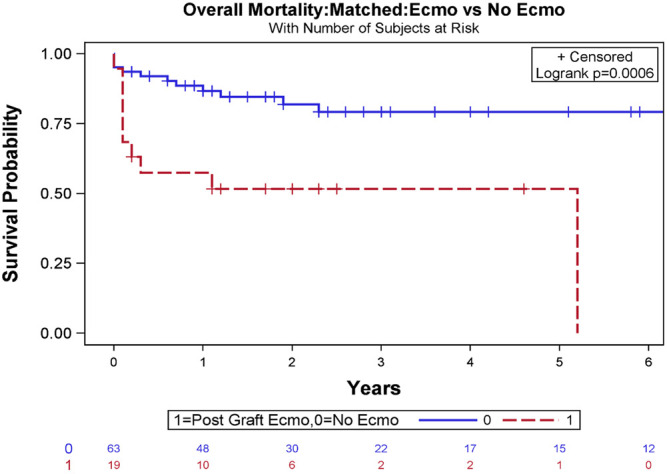
Overall survival Kaplan-Meier estimates stratified based on the requirement of veno-arterial extracorporeal membrane oxygenation (VA-ECMO) to manage severe primary graft dysfunction (PGD) following orthotopic heart transplant (OHT) after exact matching analysis (log-rank test, *p* = .0006).

For operative variables, the matched cohort without ECMO showed no significant difference compared to the ECMO cohort with regard to cardiopulmonary bypass time (*p* = .3873) and aortic cross-clamp time (*p* = .1168, Tables 3, 4). In the ECMO cohort, 30-day, 1-year, and overall survival after transplant were 73.7%, 57.9%, and 47.4%, respectively, while in the matched cohort without ECMO, 30-day, 1-year, and overall survival after transplant was 93.7%, 87.3%, and 74.6 (log-rank test, *p* = .0006, Figure 4).

**TABLE 4:** Outcomes stratified by recipient post-transplant ECMO usage, before and after exact matching.

Outcome	Before matching					After matching^a^				
	Without ECMO		With ECMO		*p*-value	Without ECMO		With ECMO		*p*-value
	*N* ^b^	Estimate	*N* ^b^	Estimate		*N* ^b^	Estimate	*N* ^b^	Estimate	
Follow up duration (years)										
Mean ± SD	880	5.66 ± 5.19	19	1.31 ± 1.53	.0005	63	3.57 ± 4.29	19	1.31 ± 1.53	.1340
Median (IQR)		4.1 (1, 9.15)		1.1 (0.1, 2)			1.9(1, 4.2)		1.1 (0.1, 2)	
Length of hospital stay (days)										
Mean ± SD	669	20.76 ± 24.35	17	49.53 ± 57.82	.0002	52	23.48 ± 27.48	17	49.53 ± 57.82	.0018
Median (IQR)		13 (10, 20)		30 (25, 39)			15.5 (11, 26)		30 (25, 39)	
Length of ICU stay (days)										
Mean ± SD	435	8.77 ± 12.74	17	37.06 ± 45.57	.0001	48	11.63 ± 17.96	17	37.06 ± 45.57	<.0001
Median (IQR)		5 (4, 8)		21 (18, 28)			6 (4, 9.5)		21 (18, 28)	
Major morbidity										
Pneumonia, *n* (%)	880	65 (7.39%)	19	6 (31.58%)	.0023	63	6 (9.52%)	19	6 (31.58%)	.0271
Urinary tract infection, *n* (%)		40 (4.55%)		0 (0%)	N/A^c^		3 (4.76%)		0 (0%)	N/A^c^
Septicemia, *n* (%)		26 (2.95%)		2 (10.53%)	.1155		2 (3.17%)		2 (10.53%)	.2281
Sternal wound infection, *n* (%)		17 (1.93%)		1 (5.26%)	.3217		2 (3.17%)		1 (5.26%)	.5516
Renal failure requiring dialysis, *n* (%)		125 (14.2%)		13 (68.42%)	<.0001		8 (12.7%)		13 (68.42%)	<.0001
Stroke, *n* (%)		3 (0.34%)		0 (0%)	N/A^c^		0 (0%)		0 (0%)	N/A^c^
Rejection within 1-yr post transplant, *n* (%)		103 (11.7%)		1 (5.26%)	.7142		7 (11.11%)		1 (5.26%)	.6740
Mortality										
30-day, *n* (%)	880	36 (4.09%)	19	5 (26.32%)	.0011	63	4 (6.35%)	19	5 (26.32%)	.0277
1-year, *n* (%)		99 (11.25%)		8 (42.11%)	.0008		8 (12.7%)		8 (42.11%)	.0084
Overall, *n* (%)		287 (32.61%)		10 (52.63%)	.0836		16 (25.4%)		10 (52.63%)	.0465

ECMO, extracorporeal membrane oxygenation.

a Patients were matched on Transplant Year (±5 years), Recipient’s Age (±4 years old), Recipient’s Gender, Recipient’s History of Prior Cardiac Surgery, and Recipient’s Preoperative Life Support (inotropic support) with those with ECMO.

b Available number of patients.

c Statistic is not applicable.

## Discussion

This comprehensive study investigated the impact of post-transplant VA-ECMO usage on the outcome of adult primary OHT recipients using the Stanford University heart transplant database. We stratified the cohort by disjoint categories of VA-ECMO usage in the early post-transplant period due to severe PGD. Severe PGD was defined as the requirement for mechanical circulatory assistance for treatment according to the ISHLT Registry consensus statement (6).

Historically, many treatments have been developed for patients with end-stage heart failure, among which OHT remains the gold standard (2). However, the persistent and worsening shortage of available donor organs has resulted in an ever-increasing waitlist of patients and longer waiting periods for heart transplants. Approximately 10% of all candidates on the waiting list for solid-organ transplantation die each year without receiving an organ (7). In order to address this challenge, we have previously proposed alternative approaches to maximize organ allocation by utilizing marginally acceptable organs (8), harvesting donor hearts from distant locations and accepting longer cold ischemic time (9), as well as utilizing obese donor hearts (10). Despite growing evidence supporting the safety of using these marginal organs, there may be concerns regarding the occurrence of PGD. Therefore, the utilization of VA-ECMO following OHT is expected to increase in the future and may become a common therapeutic option for post-transplant recipients with severely depressed postoperative cardiac output and dysfunction (3–5). Favorable outcomes of post-transplant ECMO utilization have been reported (4, 11–13). Together with improvements in technology and management of ECMO (14), these positive outcomes may also be due in part to a new approach of placing recipients with global myocardial dysfunction on ECMO rather than introducing high doses of inotropes and vasopressors.

In the current study, our data revealed that the cohort with post-transplant ECMO usage had a higher incidence of previous cardiac surgery and diabetes mellitus. In addition, our data showed a higher percentage of preoperative amiodarone and calcium channel blocker use in the ECMO cohort. Together with the previous report that pre-transplant amiodarone use is independently associated with increased incidence of severe PGD (15), we speculate that preoperative amiodarone and calcium channel blocker use may induce temporary arrhythmogenic or vasoplegia-related hemodynamic instability leading to ECMO usage following OHT, due to the effects of long-term use or overdosing of these medications. VA-ECMO can be a good treatment option to stabilize the patient until recovering from hemodynamic instability that may be related to atrioventricular conduction or vascular tone issues. In addition, our data demonstrated that a higher incidence of postoperative blood transfusion and reoperation for bleeding or tamponade was observed in recipients receiving post-transplant ECMO. We speculate that patients with previous complicated cardiac surgery are likely to have a higher chance of reoperation for bleeding or tamponade, as well as increased postoperative blood transfusion requirements. It is also possible that ECMO itself can worsen coagulopathy and cause bleeding, which eventually may require blood products, and altogether these effects may have deleterious consequences, including hemodynamic instability and PGD. This possibility is supported by reports showing that post-transplant survival was negatively affected by complications after previous placement of a VAD (16). Moreover, our data revealed that recipients undergoing ECMO following transplant had longer aortic cross-clamp time in unmatched cohort, and a previous study suggested that aortic cross-clamp time was inversely related to post-transplant survival (9).

Equally important in this study was the identification of factors that were not significantly different in the recipients’ baseline characteristics. These included the incidence of mechanical circulatory support usage, the incidence of pre-transplant hospitalization in the ICU, and donor characteristics such as age, sex, and medical history. Interestingly, our data also showed that the donor left ventricular ejection fraction was excellent in both groups. Although, in general, the perception was that heart grafts from marginal donors are of inferior quality, the incidence of post-ECMO usage due to severe PGD was observed equally regardless of recipient clinical status and donor graft quality.

Next, we discovered that the rate of severe PGD was as low as 2.1% in our cohort who underwent OHT over the last 20 years, ranking among the lowest incidences of severe PGD reported in previous studies (2–26%) (3–5, 17, 18). Although our sample size was small, we believe that the low rate of severe PGD may be attributed to our multidisciplinary patient management during the perioperative period. There may also be a number of mitigating factors related to operative techniques. Briefly, we routinely provide sufficient reperfusion time (30–240 min) together with maintaining mean arterial pressure at 75–90 mmHg on cardiopulmonary bypass, which can potentially enable the graft to recover from the stressful and edematous state and regain cardiac function following organ procurement and transplantation. This is a possible explanation for our data showing a significantly prolonged cardiopulmonary bypass time in the cohort with ECMO. We have several therapeutic options, such as leaving the chest open to remove potential mechanical stress, or aggressively introducing continuous renal replacement therapy to attenuate right ventricular dysfunction (which was reflected by our data indicating that 68.4% of the ECMO cohort required continuous renal replacement therapy). As a result of these interventions, only 2.1% required post-transplant ECMO therapy in our study cohort. Interestingly, our data did not show any statistical significance in the allograft ischemic time. This is likely because we have modified the sequence of anastomoses if the allograft ischemic time is expected to be prolonged (9).

Last, VA-ECMO can be administered using multiple techniques, including peripherally or centrally (19). Both techniques carry attendant risks of bleeding, and peripheral cannulation has an additional risk of limb ischemia. The peripheral cannulation technique, however, is minimally invasive, is immediately available, and allows rapid cannula insertion at the bedside. Femorally cannulated VA-ECMO can be discontinued without reopening the chest, which may reduce the risk of infection and re-bleeding. In the femorally cannulated VA-ECMO patients in this study, a reperfusion cannula was routinely used, and no instances of leg ischemia were observed. In the current study, two patients (10.5%) had septicemia and one patient (5.3%) had sternal wound infection in the post-transplant ECMO cohort. Given that the complications of VA-ECMO therapy increase with time, it is important to minimize the duration of VA-ECMO support. Our data showed that there were no ECMO-associated bleeding complications at the cannulation site, which is likely because our cohort had a median duration of only 7 days on ECMO support. We routinely combined IABP support for the treatment of severe PGD requiring VA-ECMO therapy. In our cohort, nine patients (56.3%) had IABP placement in addition to ECMO support. Combined IABP with ECMO therapy can additionally improve coronary perfusion and provide peripheral pulsatility, reducing left ventricular afterload by slight venting, and thereby indirectly reducing pulmonary stasis and right ventricular afterload. No IABP-associated complications were observed in our cohort. Due to the short duration of ECMO support, these patients were left intubated. Importantly, the demonstration of equivalent graft outcomes in the cohort of post-transplant ECMO survivors in adults should lower the threshold for the utilization of ECMO for severe PGD.

### Limitations of the Database

This study has limitations consistent with retrospective analyses and the use of a single-center database. The number of patients and events in each group was low, thus limiting its statistical power. The 100% follow-up and additional data, otherwise unavailable to national or international registries, are the two most important strengths of this study. The main focus of our current study is to determine the influence of post-transplant usage of ECMO on the outcome of recipients; however, specific donor or recipient characteristics may contribute to recipient mortality, and several of those have not been included in our analysis. The selection of a suitable donor is a complicated process. Clinicians need to consider multiple factors, including recipient urgency against donor characteristics, ischemic time, recipient sensitization, and donor/recipient size mismatch. Therefore, our findings may not be applicable to other centers. Only donors whose hearts were accepted for transplant were included in this study. To ascertain the real burden of marginal donors, it will be essential to distinguish donor hearts initially rejected by other centers for non-quality reasons or quality reasons (20). In addition, as this study addressed only mortality, further data are needed on the impact of post-transplant ECMO usage on morbidity in OHT. In the future, multicenter studies including larger cohorts are required.

## Conclusion

Our data suggest that VA-ECMO may be a useful salvage therapy for adult heart transplant recipients with severe PGD, especially in the setting of prior cardiac surgery history or relatively suboptimal recipient selection. In particular, the improvement in conditional survival suggests that ECMO utilization following OHT can potentially increase the use of marginally acceptable donor grafts, thereby ameliorating the shortage of donor organs, reducing waitlist times for heart transplantation, and potentially decreasing mortality rates for patients on the waiting list.

## Data Availability

The original contributions presented in the study are included in the article/supplementary material, further inquiries can be directed to the corresponding author.
